# Supplementary material for: Explicit solution of divide-and-conquer dividing by a half recurrences with polynomial independent term
Source: PLoS One. 2022 Nov 17;17(11):e0274448. doi: 10.1371/journal.pone.0274448 (PMC9671444; doi:10.1371/journal.pone.0274448)
Supplement: S1 File — The file provides the detailed proofs of Lemmas 4–6 and Propositions 9 and 12. (PDF) [file pone.0274448.s001.pdf]

Explicit solution of divide-and-conquer dividing by a half recurrences with polynomial independent term. Supplementary file S1: Proofs of several results

Tomás M. Coronado, Arnau Mir, Francesc Rosselló

This supplementary document contains the proofs of Lemmas 3–5 and Propositions 7, 10 in the main text. We freely use the notations introduced in the main text, and the equation numbering continues that of the main text.

Lemma 3. *For every $d \in \mathbb{N}$, $n \in \mathbb{N}_{\geqslant 1}$, and $x \in \mathbb{R} \setminus \{0\}$:*

(a) $\displaystyle\sum_{k=1}^{n-1} q_{s_k}(k)^d x^{q_{s_k}(k)} = T(d, q_{s_n}(n), 2x) + nq_{s_n}(n)^d x^{q_{s_n}(n)} - q_{s_n}(n)^d (2x)^{q_{s_n}(n)}$

(b) $\displaystyle\sum_{k=0}^{n-1} \sum_{i=1}^{s_k} q_i(k)^d x^{q_i(k)} = \sum_{i=1}^{s_n} 2^{q_i(n)-1} T(d, q_i(n), x) + \sum_{i=1}^{s_n} q_i(n)^d x^{q_i(n)}(n - M_i(n))$

Proof. As to (a):

$$\begin{aligned}\sum_{k=1}^{n-1} q_{s_k}(k)^d x^{q_{s_k}(k)} &= \sum_{k=1}^{2^{q_{s_n}(n)}-1} q_{s_k}(k)^d x^{q_{s_k}(k)} + \sum_{k=2^{q_{s_n}(n)}}^{n-1} q_{s_k}(k)^d x^{q_{s_k}(k)}\\ &= \sum_{j=0}^{q_{s_n}(n)-1} j^d 2^j x^j + (n - 2^{q_{s_n}(n)}) q_{s_n}(n)^d x^{q_{s_n}(n)}\end{aligned}$$

Let us prove now (b). Let $x \in \mathbb{R} \setminus \{0\}$. For every $d \in \mathbb{N}$ and $n \in \mathbb{N}_{\geqslant 1}$, set

$$a_n^{(d)} = \sum_{k=0}^{n-1} \sum_{i=1}^{s_k} q_i(k)^d x^{q_i(k)}.$$

Then,

$$\begin{aligned}a_n^{(d)} &= \sum_{k=0}^{M_2(n)-1} \sum_{i=1}^{s_k} q_i(k)^d x^{q_i(k)} + \sum_{k=M_2(n)}^{M_2(n)+2^{q_1(n)}-1} \sum_{i=1}^{s_k} q_i(k)^d x^{q_i(k)}\\ &= a_{M_2(n)}^{(d)} + \sum_{p=0}^{2^{q_1(n)}-1} \sum_{i=1}^{s_{M_2(n)+p}} q_i(M_2(n)+p)^d x^{q_i(M_2(n)+p)}\\ &= a_{M_2(n)}^{(d)} + \sum_{p=0}^{2^{q_1(n)}-1} \Big(\sum_{i=1}^{s_p} q_i(p)^d x^{q_i(p)} + \sum_{i=1}^{s_{M_2(n)}} q_i(M_2(n))^d x^{q_i(M_2(n))} \Big)\\ &= a_{M_2(n)}^{(d)} + \sum_{p=0}^{2^{q_1(n)}-1} \sum_{i=1}^{s_p} q_i(p)^d x^{q_i(p)} + 2^{q_1(n)} \sum_{i=2}^{s_n} q_i(n)^d x^{q_i(n)}\\ &= a_{M_2(n)}^{(d)} + a_{2^{q_1(n)}}^{(d)} + 2^{q_1(n)} \sum_{i=2}^{s_n} q_i(n)^d x^{q_i(n)}.\end{aligned}$$

So, a simple argument by induction shows that, for every $2 \leqslant r \leqslant s_n$

$$a_n^{(d)} = a_{M_r(n)}^{(d)} + \sum_{j=1}^{r-1} a_{2^{q_j(n)}}^{(d)} + \sum_{j=1}^{r-1} \Big(2^{q_j(n)} \sum_{i=j+1}^{s_n} q_i(n)^d x^{q_i(n)} \Big).$$

Taking $r = s_n$, we obtain

$$\begin{aligned} a_n^{(d)} &= \sum_{j=1}^{s_n} a_{2^{q_j(n)}}^{(d)} + \sum_{j=1}^{s_n-1} \Big(2^{q_j(n)} \sum_{i=j+1}^{s_n} q_i(n)^d x^{q_i(n)} \Big) \\ &= \sum_{j=1}^{s_n} a_{2^{q_j(n)}}^{(d)} + \sum_{i=2}^{s_n} \Big(q_i(n)^d x^{q_i(n)} \sum_{j=1}^{i-1} 2^{q_j(n)} \Big) \\ &= \sum_{j=1}^{s_n} a_{2^{q_j(n)}}^{(d)} + \sum_{i=2}^{s_n} q_i(n)^d x^{q_i(n)} (n - M_i(n)) \end{aligned} \tag{23}$$

Now, for every $l \in \mathbb{N}$, we have that

$$\begin{aligned} a_{2^l}^{(d)} &= \sum_{k=0}^{2^l-1} \sum_{i=1}^{s_k} q_i(k)^d x^{q_i(k)} = \sum_{t=0}^{l-1} t^d \cdot x^t \cdot \#\{(k,i) \mid 0 \leqslant k \leqslant 2^l - 1,\ q_i(k) = t\} \\ &= 2^{l-1} \sum_{t=0}^{l-1} t^d \cdot x^t = 2^{l-1} T(d,l,x) \end{aligned} \tag{24}$$

because each $\#\{(k,i) \mid 0 \leqslant k \leqslant 2^l - 1,\ q_i(k) = t\}$ is simply the number of binary words $w \in \{0,1\}^l$ with an 1 in the position $t+1$ starting from the right, which is 2^{l-1}.

Using this expression in Eqn. (23), and taking into account that $M_1(n) = n$, we obtain the formula in the statement. □

In particular:

- If $x \neq 1/2$,

$$\sum_{k=1}^{n-1} x^{q_{s_k}(k)} = \frac{(2x)^{q_{s_n}(n)} - 1}{2x - 1} + n \cdot x^{q_{s_n}(n)} - (2x)^{q_{s_n}(n)}$$

$$\sum_{k=1}^{n-1} q_{s_k}(k) x^{q_{s_k}(k)} = \frac{q_{s_n}(n)(2x)^{q_{s_n}(n)}}{2x-1} - \frac{2x((2x)^{q_{s_n}(n)} - 1)}{(2x-1)^2} + n q_{s_n}(n) x^{q_{s_n}(n)} - q_{s_n}(n)(2x)^{q_{s_n}(n)}$$

- If $x = 1/2$,

$$\sum_{k=1}^{n-1} x^{q_{s_k}(k)} = q_{s_n}(n) + n \cdot 2^{-q_{s_n}(n)} - 1$$

$$\sum_{k=1}^{n-1} q_{s_k}(k) x^{q_{s_k}(k)} = \frac{q_{s_n}(n)(q_{s_n}(n) - 3)}{2} + n q_{s_n}(n) 2^{-q_{s_n}(n)}$$

- If $x \neq 1$,

$$\sum_{k=0}^{n-1} \sum_{i=1}^{s_k} x^{q_i(k)} = \sum_{i=1}^{s_n} x^{q_i(n)} (n - M_i(n)) + \sum_{i=1}^{s_n} 2^{q_i(n)} \cdot \frac{x^{q_i(n)} - 1}{2(x-1)} \tag{25}$$

$$\sum_{k=0}^{n-1} \sum_{j=1}^{s_k} q_j(k) x^{q_j(k)} = \sum_{i=1}^{s_n} q_i(n) x^{q_i(n)} (n - M_i(n)) + \sum_{i=1}^{s_n} 2^{q_i(n)} \cdot \frac{(x-1) q_i(n) x^{q_i(n)} - x^{q_i(n)+1} + x}{2(x-1)^2} \tag{26}$$

- If $x = 1$,

$$\sum_{k=0}^{n-1} s_k = \sum_{k=1}^{n-1} \sum_{i=1}^{s_k} 1^{q_i(k)} = \sum_{i=1}^{s_n} 2^{q_i(n)-1}(q_i(n) + 2(s_n - i)) \tag{27}$$

$$\sum_{k=1}^{n-1} \sum_{j=1}^{s_k} q_j(k) = \sum_{i=1}^{s_n} 2^{q_i(n)-1}\Big(\binom{q_i(n)}{2} + 2\sum_{j=i+1}^{s_n} q_j(n)\Big) \tag{28}$$

Lemma 4. *Let $(z_{l,p})_{(l,p)\in\mathbb{N}^2}$ be a double sequence satisfying:*

(a) For every $p \in \mathbb{N}$, $z_{0,p} = 0$

(b) For every $l, p > 0$,

$$z_{l,p} = 2z_{l-1,p} + \sum_{q=0}^{p-1} \binom{p}{q} 2^{(p-q)(l-1)} z_{l-1,q}$$

Then, for every $l \geqslant 0$ and for every $p > 0$,

$$z_{l,p} = -\frac{2^l}{p+1} \sum_{j=1}^{l-1} \Big(\sum_{i=1}^{p} \binom{p+1}{i} (2^i - 1) 2^{(p-i)l+(i-1)j} B_i\Big) z_{j,0}.$$

Proof. Let us iterate several times the recurrence defining our double sequence:

$$\begin{aligned}
z_{l,p} &= 2z_{l-1,p} + \sum_{q=0}^{p-1} \binom{p}{q} 2^{(p-q)(l-1)} z_{l-1,q} \\
&= 2\Big(2z_{l-2,p} + \sum_{q=0}^{p-1} \binom{p}{q} 2^{(p-q)(l-2)} z_{l-2,q}\Big) + \sum_{q=0}^{p-1} \binom{p}{q} 2^{(p-q)(l-1)} z_{l-1,q} \\
&= 2^2 z_{l-2,p} + \sum_{q=0}^{p-1} \binom{p}{q} \Big(2^{(p-q)(l-2)+1} z_{l-2,q} + 2^{(p-q)(l-1)} z_{l-1,q}\Big) \\
&= 2^2 \Big(2z_{l-3,p} + \sum_{q=0}^{p-1} \binom{p}{q} 2^{(p-q)(l-3)} z_{l-3,q}\Big) + \sum_{q=0}^{p-1} \binom{p}{q} \Big(2^{(p-q)(l-2)+1} z_{l-2,q} + 2^{(p-q)(l-1)} z_{l-1,q}\Big) \\
&= 2^3 z_{l-3,p} + \sum_{q=0}^{p-1} \binom{p}{q} \Big(2^{(p-q)(l-3)+2} z_{l-3,q} + 2^{(p-q)(l-2)+1} z_{l-2,q} + 2^{(p-q)(l-1)} z_{l-1,q}\Big)
\end{aligned}$$

Reasoning in this way, and recalling that $z_{0,q} = 0$ for every q, it is easy to prove by induction on l that

$$z_{l,p} = \sum_{q=0}^{p-1} \binom{p}{q} \Big(\sum_{j=1}^{l-1} 2^{(p-q)j+(l-1-j)} z_{j,q}\Big) = 2^{l-1} \sum_{q=0}^{p-1} \binom{p}{q} \Big(\sum_{j=1}^{l-1} 2^{(p-q-1)j} z_{j,q}\Big) \tag{29}$$

We have computed explicitly $z_{l,p}$ for several $p \geqslant 1$ using this recurrence, in order to look for a pattern. The

results have been:

$$
\begin{aligned}
z_{l,2} =& 2^{l-1}\sum_{j=1}^{l-1}(2^l - 2\cdot 2^{j-1})z_{j,0}\\
z_{l,3} =& 2^{l-1}\sum_{j=1}^{l-1}\left(2^{2l} - 3\cdot 2^{l+j-1}\right)z_{j,0}\\
z_{l,4} =& 2^{l-1}\sum_{j=1}^{l-1}\left(2^{3l} - 4\cdot 2^{2l+j-1} + 8\cdot 2^{3(j-1)}\right)z_{j,0}\\
z_{l,5} =& 2^{l-1}\sum_{j=1}^{l-1}(2^{4l} - 5\cdot 2^{3l+j-1} + 20\cdot 2^{l+3(j-1)})z_{j,0}\\
z_{l,6} =& 2^{l-1}\sum_{j=1}^{l-1}(2^{5l} - 6\cdot 2^{4l+j-1} + 40\cdot 2^{2l+3(j-1)} - 96\cdot 2^{5(j-1)})z_{j,0}\\
z_{l,7} =& 2^{l-1}\sum_{j=1}^{l-1}(2^{6l} - 7\cdot 2^{5l+j-1} + 70\cdot 2^{3l+3(j-1)} - 336\cdot 2^{l+5(j-1)})z_{j,0}\\
z_{l,8} =& 2^{l-1}\sum_{j=1}^{l-1}(2^{7l} - 8\cdot 2^{6l+j-1} + 112\cdot 2^{4l+3(j-1)} - 896\cdot 2^{2l+5(j-1)} + 2176\cdot 2^{7(j-1)})z_{j,0}\\
z_{l,9} =& 2^{l-1}\sum_{j=1}^{l-1}(2^{8l} - 9\cdot 2^{7l+j-1} + 168\cdot 2^{5l+3(j-1)} - 2016\cdot 2^{3l+5(j-1)} + 9792\cdot 2^{l+7(j-1)})z_{j,0}\\
z_{l,10} =& 2^{l-1}\sum_{j=1}^{l-1}(2^{9l} - 10\cdot 2^{8l+j-1} + 240\cdot 2^{6l+3(j-1)} - 4032\cdot 2^{4l+5(j-1)} + 32640\cdot 2^{2l+7(j-1)}\\
& \qquad - 79360\cdot 2^{9(j-1)})z_{j,0}\\
z_{l,11} =& 2^{l-1}\sum_{j=1}^{l-1}(2^{10l} - 11\cdot 2^{9l+j-1} + 330\cdot 2^{7l+3(j-1)} - 7392\cdot 2^{5l+5(j-1)} + 89760\cdot 2^{3l+7(j-1)}\\
& \qquad - 436480\cdot 2^{l+9(j-1)})z_{j,0},\\
z_{l,12} =& 2^{l-1}\sum_{j=1}^{l-1}(2^{11l} - 12\cdot 2^{10l+j-1} + 440\cdot 2^{8l+3(j-1)} - 12672\cdot 2^{6l+5(j-1)} + 215424\cdot 2^{4l+7(j-1)}\\
& \qquad - 1745920\cdot 2^{2l+9(j-1)} + 4245504\cdot 2^{11(j-1)})z_{j,0}.
\end{aligned}
$$

These results hint that

$$z_{l,p} = 2^{l-1}\sum_{j=1}^{l-1}\left(2^{(p-1)l} + \sum_{i=1}^{p-1} a_i^{(p)}\binom{p}{i}2^{(p-1-i)l+i(j-1)}\right)z_{j,0}.$$

with $a_{2i}^{(p)} = 0$ if $i > 0$,

$$a_1^{(p)} = -1,\ a_3^{(p)} = 2,\ a_5^{(p)} = -16,\ a_7^{(p)} = 272,\ a_9^{(p)} = -7936, \ldots$$

These values are consistent with

$$a_i^{(p)} = -\frac{1}{i+1}\cdot 2^{i+1}(2^{i+1}-1)B_{i+1}, \quad \text{for } 1 \leqslant i \leqslant p-1$$

So, this leads us to conjecture that, if $p > 0$,

$$
\begin{aligned}
z_{l,p} &= 2^{l-1}\sum_{j=1}^{l-1}\Big(2^{(p-1)l} - \sum_{i=1}^{p-1}\frac{1}{i+1}\cdot 2^{i+1}(2^{i+1}-1)B_{i+1}\binom{p}{i}2^{(p-1-i)l+i(j-1)}\Big)z_{j,0}\\
&= 2^{l-1}\sum_{j=1}^{l-1}\Big(2^{(p-1)l} - \sum_{i=1}^{p-1}\frac{1}{p+1}\cdot 2^{i+1}(2^{i+1}-1)B_{i+1}\binom{p+1}{i+1}2^{(p-1-i)l+i(j-1)}\Big)z_{j,0}\\
&= 2^{l-1}\sum_{j=1}^{l-1}\Big(2^{(p-1)l} - \sum_{i=2}^{p}\frac{1}{p+1}\cdot 2^{i}(2^{i}-1)B_{i}\binom{p+1}{i}2^{(p-i)l+(i-1)(j-1)}\Big)z_{j,0}\\
&= 2^{l-1}\sum_{j=1}^{l-1}\Big(2^{(p-1)l} - \sum_{i=1}^{p}\frac{1}{p+1}\cdot 2^{i}(2^{i}-1)B_{i}\binom{p+1}{i}2^{(p-i)l+(i-1)(j-1)}\\
&\qquad + \frac{1}{p+1}\cdot 2B_1(p+1)2^{(p-1)l}\Big)z_{j,0}\\
&= 2^{l-1}\sum_{j=1}^{l-1}\Big(2^{(p-1)l} - \sum_{i=1}^{p}\frac{1}{p+1}\cdot 2^{i}(2^{i}-1)B_{i}\binom{p+1}{i}2^{(p-i)l+(i-1)(j-1)} - 2^{(p-1)l}\Big)z_{j,0}\\
&= -\frac{2^l}{p+1}\sum_{j=1}^{l-1}\Big(\sum_{i=1}^{p}(2^i-1)\binom{p+1}{i}2^{(p-i)l+(i-1)j}B_i\Big)z_{j,0} \qquad (30)
\end{aligned}
$$

and we proceed to prove this equality by induction on l.

The case when $l = 1$ is true because the right hand sum of (30) is empty and, by (29), $z_{1,p} = 0$. Assume now that the equality (30) is true for $z_{1,p}, \dots, z_{l-1,p}$ and every $p > 0$, and let us prove it for $z_{l,p}$. By (29) and the induction hypothesis,

$$
\begin{aligned}
2^{1-l}z_{l,p} &= \sum_{q=0}^{p-1}\binom{p}{q}\sum_{k=1}^{l-1}2^{(p-q-1)k}z_{k,q}\\
&= \sum_{k=1}^{l-1}2^{(p-1)k}z_{k,0}\\
&\quad -\sum_{q=1}^{p-1}\binom{p}{q}\sum_{k=1}^{l-1}2^{(p-q-1)k}\cdot 2^{k-1}\sum_{j=1}^{k-1}\Big(\sum_{i=1}^{q}\frac{2^i(2^i-1)B_i}{q+1}\binom{q+1}{i}2^{(q-i)k+(i-1)(j-1)}\Big)z_{j,0}\\
&\quad \text{(by the induction hypothesis)}\\
&= \sum_{j=1}^{l-1}2^{(p-1)j}z_{j,0}\\
&\quad -\sum_{j=1}^{l-2}\left[\sum_{q=1}^{p-1}\binom{p}{q}\sum_{k=j+1}^{l-1}2^{(p-q)k-1}\sum_{i=1}^{q}\frac{2^i(2^i-1)B_i}{q+1}\binom{q+1}{i}2^{(q-i)k+(i-1)(j-1)}\right]z_{j,0}\\
&= \sum_{j=1}^{l-1}\left[2^{(p-1)j}-\sum_{q=1}^{p-1}\binom{p}{q}\sum_{k=j+1}^{l-1}2^{(p-q)k-1}\sum_{i=1}^{q}\frac{2^i(2^i-1)B_i}{q+1}\binom{q+1}{i}2^{(q-i)k+(i-1)(j-1)}\right]z_{j,0}
\end{aligned}
$$

and then the coefficient of $z_{j,0}$, for $j \leqslant l-1$, in $2^{-l+1}z_{l,p}$, which we want to prove to be

$$
-\frac{2}{p+1}\sum_{i=1}^{p}(2^i-1)B_i\binom{p+1}{i}2^{(p-i)l+(i-1)j}
$$

is

$$
\begin{aligned}
&2^{(p-1)j} - \sum_{q=1}^{p-1} \binom{p}{q} \sum_{k=j+1}^{l-1} 2^{(p-q)k-1} \sum_{i=1}^{q} \frac{2^i(2^i-1)B_i}{q+1} \binom{q+1}{i} 2^{(q-i)k+(i-1)(j-1)} \\
&= 2^{(p-1)j} - \sum_{i=1}^{p-1}\sum_{q=i}^{p-1}\sum_{k=j+1}^{l-1} \frac{(2^i-1)B_i}{q+1} \binom{p}{q}\binom{q+1}{i} 2^{(p-i)k+(i-1)j} \\
&= 2^{(p-1)j} - \sum_{i=1}^{p-1}\sum_{q=i}^{p-1}\sum_{k=j+1}^{l-1} \frac{(2^i-1)B_i}{p+1} \binom{p+1}{q+1}\binom{q+1}{i} 2^{(p-i)k+(i-1)j} \\
&= 2^{(p-1)j} - \sum_{i=1}^{p-1}\sum_{q=i}^{p-1}\sum_{k=j+1}^{l-1} \frac{(2^i-1)B_i}{p+1} \binom{p+1}{i}\binom{p+1-i}{q+1-i} 2^{(p-i)k+(i-1)j} \\
&= 2^{(p-1)j} - \sum_{i=1}^{p-1} \frac{2^{(i-1)j}(2^i-1)B_i}{p+1} \binom{p+1}{i} \Big(\sum_{q=i}^{p-1} \binom{p+1-i}{q+1-i}\Big)\Big(\sum_{k=j+1}^{l-1} 2^{(p-i)k}\Big) \\
&= 2^{(p-1)j} - \sum_{i=1}^{p-1} \frac{2^{(i-1)j}(2^i-1)B_i}{p+1} \binom{p+1}{i} 2(2^{p-i}-1) \frac{2^{(p-i)l} - 2^{(p-i)(j+1)}}{2^{p-i}-1} \\
&= 2^{(p-1)j} - \sum_{i=1}^{p-1} \frac{2(2^i-1)B_i}{p+1} \binom{p+1}{i} 2^{(i-1)j}\big(2^{(p-i)l} - 2^{(p-i)(j+1)}\big) \\
&= 2^{(p-1)j} - \sum_{i=1}^{p-1} \frac{2(2^i-1)B_i}{p+1} \binom{p+1}{i} 2^{(p-i)l+(i-1)j} + \sum_{i=1}^{p-1} \frac{2(2^i-1)B_i}{p+1} \binom{p+1}{i} 2^{p-i+(p-1)j} \\
&= 2^{(p-1)j} - \sum_{i=0}^{p} \frac{2(2^i-1)B_i}{p+1} \binom{p+1}{i} 2^{(p-i)l+(i-1)j} + \sum_{i=0}^{p} \frac{2(2^i-1)B_i}{p+1} \binom{p+1}{i} 2^{p-i+(p-1)j} \\
&= 2^{(p-1)j} - \sum_{i=0}^{p} \frac{2(2^i-1)B_i}{p+1} \binom{p+1}{i} 2^{(p-i)l+(i-1)j} + \frac{2^{p+1+(p-1)j}}{p+1} \sum_{i=0}^{p} \binom{p+1}{i} B_i \\
&\qquad - \frac{2^{(p-1)j}}{p+1} \sum_{i=0}^{p} \binom{p+1}{i} 2^{p+1-i} B_i \\
&= 2^{(p-1)j} - \sum_{i=0}^{p} \frac{2(2^i-1)B_i}{p+1} \binom{p+1}{i} 2^{(p-i)l+(i-1)j} - \frac{2^{(p-1)j}}{p+1} \big(B_{p+1}(2) - B_{p+1}\big) \\
&= - \sum_{i=0}^{p} \frac{2(2^i-1)B_i}{p+1} \binom{p+1}{i} 2^{(p-i)l+(i-1)j} \quad \text{(by Eqn. (11))}
\end{aligned}
$$

as we wanted to prove. □

Lemma 5. *For every $l, m, p, d \in \mathbb{N}$, with $p < m$,*

$$
\begin{aligned}
\gamma_l^{(d,p,m)} = \frac{a^{l-1} 2^{-(m-1)(l-1)+pl}}{p+1} \sum_{t=1}^{l-1} (l-t-1)^d (a^{-1}2^{m-p-1})^t (B_{p+1}(2^t) - B_{p+1}) \\
+ (a2^{-(m-1)})^{l-1} (l-1)^d \cdot \delta_{p=0,l>0}
\end{aligned}
$$

where $\delta_{p=0,l>0} = 1$ if $p = 0$ and $l > 0$, and $\delta_{p=0,l>0} = 0$ otherwise.

Proof. When $l = 0$, both sides of the equality in the statement are equal to 0. When $l, p > 0$,

$$\begin{aligned}
\gamma_l^{(d,p,m)} &= \gamma_{l-1}^{(d,p,m)} + \sum_{k=2^{l-1}}^{2^l-1} \sum_{i=1}^{s_k} q_i(k)^d (a2^{-m})^{q_i(k)} M_{i+1}(k)^p \\
&= \gamma_{l-1}^{(d,p,m)} + \sum_{k=2^{l-1}}^{2^l-1} \sum_{i=1}^{s_k} q_i(k)^d (a2^{-m})^{q_i(k)} \Big(M_{i+1}(k - 2^{l-1}) + 2^{l-1} \Big)^p \\
&= \gamma_{l-1}^{(d,p,m)} + \sum_{k=0}^{2^{l-1}-1} \sum_{i=1}^{s_k} q_i(k)^d (a2^{-m})^{q_i(k)} \Big(M_{i+1}(k) + 2^{l-1} \Big)^p \\
&= \gamma_{l-1}^{(d,p,m)} + \sum_{k=1}^{2^{l-1}-1} \sum_{i=1}^{s_k} q_i(k)^d (a2^{-m})^{q_i(k)} \sum_{q=0}^{p} \binom{p}{q} M_{i+1}(k)^q 2^{(p-q)(l-1)} \\
&= \gamma_{l-1}^{(d,p,m)} + \sum_{q=0}^{p} \binom{p}{q} 2^{(p-q)(l-1)} \sum_{k=1}^{2^{l-1}-1} \sum_{i=1}^{s_k} q_i(k)^d (a2^{-m})^{q_i(k)} M_{i+1}(k)^q \\
&= \gamma_{l-1}^{(d,p,m)} + \sum_{q=0}^{p} \binom{p}{q} 2^{(p-q)(l-1)} \gamma_{l-1}^{(d,q,m)} = 2\gamma_{l-1}^{(d,p,m)} + \sum_{q=0}^{p-1} \binom{p}{q} 2^{(p-q)(l-1)} \gamma_{l-1}^{(d,q,m)}
\end{aligned}$$

Then, by Lemma 4 and Eqn. (15) in the main text,

$$\begin{aligned}
\gamma_l^{(d,p,m)} &= -\frac{2^l}{p+1} \sum_{j=1}^{l-1} \Big(\sum_{i=1}^{p} \binom{p+1}{i} (2^i - 1) 2^{(p-i)l + (i-1)j} B_i \Big) \gamma_j^{(d,0,m)} \\
&= -\frac{2^l}{p+1} \sum_{j=1}^{l-1} \left[\sum_{i=1}^{p} \binom{p+1}{i} (2^i - 1) 2^{(p-i)l+(i-1)j} B_i 2^{j-1} \sum_{t=0}^{j-1} t^d (a2^{-m})^t \right] \\
&= -\frac{2^{pl+l-1}}{p+1} \sum_{i=1}^{p} \left[\binom{p+1}{i} (2^i - 1) 2^{-il} \Big(\sum_{t=0}^{l-2} t^d (a2^{-m})^t \sum_{j=t+1}^{l-1} 2^{ij} \Big) \right] B_i \\
&= -\frac{2^{pl+l-1}}{p+1} \sum_{i=1}^{p} \left[\binom{p+1}{i} (2^i - 1) 2^{-il} \Big(\sum_{t=0}^{l-2} t^d (a2^{-m})^t \cdot \frac{2^{il} - 2^{i(t+1)}}{2^i - 1} \Big) \right] B_i \\
&= -\frac{2^{pl+l-1}}{p+1} \sum_{i=1}^{p} \binom{p+1}{i} \Big(\sum_{t=0}^{l-2} t^d (a2^{-m})^t \Big) B_i + \frac{2^{pl+l-1}}{p+1} \sum_{i=1}^{p} \binom{p+1}{i} 2^{-i(l-1)} \Big(\sum_{t=0}^{l-2} t^d (a2^{i-m})^t \Big) B_i \\
&= \frac{2^{pl+l-1}}{p+1} \Big(\sum_{t=0}^{l-2} t^d (a2^{-m})^t \Big) B_0 + \frac{2^{pl+l-1}}{p+1} \sum_{i=1}^{p} \binom{p+1}{i} 2^{-i(l-1)} \Big(\sum_{t=0}^{l-2} t^d (a2^{i-m})^t \Big) B_i \\
&= \frac{2^{pl+l-1}}{p+1} \sum_{i=0}^{p} \binom{p+1}{i} 2^{-i(l-1)} \Big(\sum_{t=0}^{l-2} t^d (a2^{i-m})^t \Big) B_i \\
&= \frac{2^{pl+l-1}}{p+1} \sum_{i=0}^{p} \binom{p+1}{i} 2^{-i(l-1)} \Big(\sum_{t=1}^{l-1} (l-1-t)^d (a2^{i-m})^{l-1-t} \Big) B_i \\
&= \frac{a^{l-1} 2^{-(m-1)(l-1)+pl}}{p+1} \sum_{t=1}^{l-1} (l-t-1)^d (a^{-1} 2^{m-p-1})^t \Big(\sum_{i=0}^{p} \binom{p+1}{i} 2^{(p+1-i)t} B_i \Big) \\
&= \frac{a^{l-1} 2^{-(m-1)(l-1)+pl}}{p+1} \sum_{t=1}^{l-1} (l-t-1)^d (a^{-1} 2^{m-p-1})^t (B_{p+1}(2^t) - B_{p+1})
\end{aligned}$$

This finishes the case $l, p > 0$.

Finally, when $p = 0$ and $l > 0$, by Eqn. (15),

$$\begin{aligned}\gamma_l^{(d,0,m)} &= 2^{l-1}\sum_{t=0}^{l-1} t^d (a2^{-m})^t = 2^{l-1}\sum_{t=0}^{l-1}(l-1-t)^d(a2^{-m})^{l-1-t}\\ &= a^{l-1}2^{-(m-1)(l-1)}\Big(\sum_{t=1}^{l-1}(l-1-t)^d(a^{-1}2^m)^t + (l-1)^d\Big)\\ &= a^{l-1}2^{-(m-1)(l-1)}\Big(\sum_{t=1}^{l-1}(l-1-t)^d(a^{-1}2^{m-1})^t(B_1(2^t)-B_1) + (l-1)^d\Big)\\ &= \frac{a^{l-1}2^{-(m-1)(l-1)+0\cdot l}}{0+1}\sum_{t=1}^{l-1}(l-t-1)^d(a^{-1}2^{m-0-1})^t(B_{0+1}(2^t)-B_{0+1})\\ &\quad + (a2^{-(m-1)})^{l-1}(l-1)^d\end{aligned}$$

□

Proposition 7. *For every $n \geqslant 2$,*

$$\begin{aligned} y_n^{(r,t)} = a^{q_{s_{n-1}}(n-1)} &+ \sum_{\substack{l=0\\ l\neq\ell}}^{r-1}\frac{\binom{r}{l}}{2^{t+l}-a}\big((n-1)^{t+l} - a^{q_{s_{n-1}}(n-1)}\big)\\ &+ \sum_{i=0}^{r+t-1}\Big(2^{-i}\binom{r+t}{i} - 2^{-i+1}\binom{r}{i-t} - \sum_{\substack{l=i-t+1\\ l\neq\ell}}^{r-1}\frac{\binom{r}{l}\binom{t+l}{i}}{2^{t+l}-a}\Big)S_{n-1}^{(0,i)}\\ &+ \delta_\ell\cdot\frac{1}{a}\binom{r}{\ell}\sum_{j=0}^{s_{n-1}-1} M_{j+1}(n-1)^{t+\ell}(q_{j+1}(n-1)-q_j(n-1))\end{aligned}$$

Proof. Let $n \in \mathbb{N}_{\geqslant 2}$. To simplify the notations, in this proof we denote s_{n-1} by s and each $q_i(n-1)$ and $M_i(n-1)$, $i = 1,\ldots,s$, by q_i and M_i, respectively. Recall that $\ell = \log_2(a) - t$ if $a > 0$. This number will play a role in sums of geometric sequences of the form $\sum_{k=1}^m (2^{-(t+l)}a)^k$ with $l \in \mathbb{N}$, which yield two different expressions depending on whether $l = \ell$, that is, $2^{-(t+l)}a = 1$, or not.

It is easy to check that when $n = 2$ the right-hand side in the formula for $y_n^{(r,t)}$ given in the statement is 1, using that $s_1 = 1$ and $q_{s_1}(1) = 0$. Assume now that $n \geqslant 3$. We begin by deriving a recurrence for $y_n^{(r,t)}$ when $n \geqslant 3$. Taking into account the parity of n, we have:

- For every $m \geqslant 2$,

$$\begin{aligned} y_{2m}^{(r,t)} &= x_{2m}^{(r,t)} - x_{2m-1}^{(r,t)}\\ &= 2a\cdot x_m^{(r,t)} + m^{r+t} - a\cdot x_m^{(r,t)} - a\cdot x_{m-1}^{(r,t)} - m^r(m-1)^t = a\cdot y_m^{(r,t)} + m^{r+t} - m^r(m-1)^t\\ &= a\cdot y_{\lceil(2m)/2\rceil}^{(r,t)} + \lceil(2m)/2\rceil^r\lfloor(2m)/2\rfloor^t - \lfloor(2m)/2\rfloor^r(\lceil(2m)/2\rceil-1)^t\end{aligned}$$

- For every $m \geqslant 1$,

$$\begin{aligned} y_{2m+1}^{(r,t)} &= x_{2m+1}^{(r,t)} - x_{2m}^{(r,t)}\\ &= a\cdot x_{m+1}^{(r,t)} + a\cdot x_m^{(r,t)} + (m+1)^r m^t - 2a\cdot x_m^{(r,t)} - m^{r+t} = a\cdot y_{m+1}^{(r,t)} + (m+1)^r m^t - m^{r+t}\\ &= a\cdot y_{\lceil(2m+1)/2\rceil}^{(r,t)} + \lceil(2m+1)/2\rceil^r\lfloor(2m+1)/2\rfloor^t - \lfloor(2m+1)/2\rfloor^r(\lceil(2m+1)/2\rceil-1)^t\end{aligned}$$

So, in summary, $y_n^{(r,t)}$ satisfies the recurrence

$$y_n^{(r,t)} = a \cdot y_{\varphi_1(n)}^{(r,t)} + \varphi_1(n)^r \varphi_0(n)^t - \varphi_0(n)^r (\varphi_1(n) - 1)^t, \quad n \geqslant 3.$$

Therefore, for every $n \geqslant 3$ and $m \geqslant 1$,

$$y_n^{(r,t)} = a^m \cdot y_{\varphi_{\underbrace{1\ldots1}_{m}}(n)}^{(r,t)} + \sum_{k=0}^{m-1} a^k \Big(\varphi_{1\underbrace{1\ldots1}_{k}}(n)^r \varphi_{0\underbrace{1\ldots1}_{k}}(n)^t - \varphi_{0\underbrace{1\ldots1}_{k}}(n)^r (\varphi_{1\underbrace{1\ldots1}_{k}}(n) - 1)^t \Big)$$

where, by Eqn. (6), for every $k \geqslant 0$

$$\varphi_{1\underbrace{1\ldots1}_{k}}(n) = \left\lfloor \frac{n + 2^{k+1} - 1}{2^{k+1}} \right\rfloor = \left\lfloor \frac{n-1}{2^{k+1}} \right\rfloor + 1$$

$$\varphi_{0\underbrace{1\ldots1}_{k}}(n) = \left\lfloor \frac{n + 2^{k} - 1}{2^{k+1}} \right\rfloor = \left\lfloor \frac{n-1}{2^{k+1}} + \frac{1}{2} \right\rfloor$$

This implies that, for every $n \geqslant 3$,

$$y_n^{(r,t)} = a^{L_n} y_2^{(r,t)} + \sum_{k=1}^{L_n} a^{k-1} \left(\left(1 + \left\lfloor \frac{n-1}{2^k} \right\rfloor \right)^r \left\lfloor \frac{n-1}{2^k} + \frac{1}{2} \right\rfloor^t - \left\lfloor \frac{n-1}{2^k} + \frac{1}{2} \right\rfloor^r \left\lfloor \frac{n-1}{2^k} \right\rfloor^t \right)$$

where L_n is such that $2 = \varphi_{\underbrace{1\ldots1}_{L_n}}(n) = 1 + \lfloor (n-1)/2^{L_n} \rfloor$ that is, $L_n = q_{s_{n-1}}(n-1)$, which we denote by q_s.

Thus, in summary:

$$y_n^{(r,t)} = a^{q_s} + \sum_{k=1}^{q_s} a^{k-1} \left[\left(1 + \left\lfloor \frac{n-1}{2^k} \right\rfloor \right)^r \left\lfloor \frac{n-1}{2^k} + \frac{1}{2} \right\rfloor^t - \left\lfloor \frac{n-1}{2^k} + \frac{1}{2} \right\rfloor^r \left\lfloor \frac{n-1}{2^k} \right\rfloor^t \right] \tag{31}$$

Let us compute now

$$\widehat{y}_n = \sum_{k=1}^{q_s} a^{k-1} \left(\left(1 + \left\lfloor \frac{n-1}{2^k} \right\rfloor \right)^r \left\lfloor \frac{n-1}{2^k} + \frac{1}{2} \right\rfloor^t - \left\lfloor \frac{n-1}{2^k} + \frac{1}{2} \right\rfloor^r \left\lfloor \frac{n-1}{2^k} \right\rfloor^t \right)$$

To begin with, notice that:

- If $k \leqslant q_1$,

$$\begin{aligned} &\Big(1 + \left\lfloor \frac{n-1}{2^k} \right\rfloor \Big)^r \left\lfloor \frac{n-1}{2^k} + \frac{1}{2} \right\rfloor^t - \left\lfloor \frac{n-1}{2^k} + \frac{1}{2} \right\rfloor^r \left\lfloor \frac{n-1}{2^k} \right\rfloor^t \\ &\quad = \Big(1 + \frac{n-1}{2^k}\Big)^r \Big(\frac{n-1}{2^k}\Big)^t - \Big(\frac{n-1}{2^k}\Big)^r \Big(\frac{n-1}{2^k}\Big)^t \\ &\quad = (1 + 2^{-k} M_1)^r (2^{-k} M_1)^t - (2^{-k} M_1)^{r+t} = \sum_{l=0}^{r-1} \binom{r}{l} (2^{-k} M_1)^{t+l} \end{aligned}$$

- If $q_j + 1 < k \leqslant q_{j+1}$, for some $j = 1, \ldots, s-1$,

$$\begin{aligned} &\Big(1 + \left\lfloor \frac{n-1}{2^k} \right\rfloor \Big)^r \left\lfloor \frac{n-1}{2^k} + \frac{1}{2} \right\rfloor^t - \left\lfloor \frac{n-1}{2^k} + \frac{1}{2} \right\rfloor^r \left\lfloor \frac{n-1}{2^k} \right\rfloor^t \\ &\quad = \Big(1 + \left\lfloor \sum_{i=1}^{s} 2^{q_i - k} \right\rfloor \Big)^r \left\lfloor \sum_{i=1}^{s} 2^{q_i - k} + \frac{1}{2} \right\rfloor^t - \left\lfloor \sum_{i=1}^{s} 2^{q_i - k} + \frac{1}{2} \right\rfloor^r \left\lfloor \sum_{i=1}^{s} 2^{q_i - k} \right\rfloor^t \\ &\quad = \Big(1 + \sum_{i=j+1}^{s} 2^{q_i - k}\Big)^r \Big(\sum_{i=j+1}^{s} 2^{q_i - k}\Big)^t - \Big(\sum_{i=j+1}^{s} 2^{q_i - k}\Big)^r \Big(\sum_{i=j+1}^{s} 2^{q_i - k}\Big)^t \\ &\quad = (1 + 2^{-k} M_{j+1})^r (2^{-k} M_{j+1})^t - (2^{-k} M_{j+1})^{r+t} = \sum_{l=0}^{r-1} \binom{r}{l} (2^{-k} M_{j+1})^{t+l} \end{aligned}$$

- If $k = q_j + 1$, for some $j = 1, \dots, s-1$,

$$
\begin{aligned}
&\Big(1+\Big\lfloor \frac{n-1}{2^k}\Big\rfloor\Big)^r \Big\lfloor \frac{n-1}{2^k}+\frac{1}{2}\Big\rfloor^t - \Big\lfloor \frac{n-1}{2^k}+\frac{1}{2}\Big\rfloor^r \Big\lfloor \frac{n-1}{2^k}\Big\rfloor^t \\
&= \Big(1+\Big\lfloor \sum_{i=1}^{s} 2^{q_i-q_j-1}\Big\rfloor\Big)^r \Big\lfloor \sum_{i=1}^{s} 2^{q_i-q_j-1}+\frac{1}{2}\Big\rfloor^t - \Big\lfloor \sum_{i=1}^{s} 2^{q_i-q_j-1}+\frac{1}{2}\Big\rfloor^r \Big\lfloor \sum_{i=1}^{s} 2^{q_i-q_j-1}\Big\rfloor^t \\
&= \Big(1+\sum_{i=j+1}^{s} 2^{q_i-q_j-1}\Big)^r \Big(\sum_{i=j+1}^{s} 2^{q_i-q_j-1}+1\Big)^t \\
&\quad - \Big(\sum_{i=j+1}^{s} 2^{q_i-q_j-1}+1\Big)^r \Big(\sum_{i=j+1}^{s} 2^{q_i-q_j-1}\Big)^t \\
&= (1+2^{-q_j-1}M_{j+1})^{r+t} - (1+2^{-q_j-1}M_{j+1})^r (2^{-q_j-1}M_{j+1})^t \\
&= \sum_{i=0}^{r+t-1} \binom{r+t}{i} (2^{-q_j-1}M_{j+1})^i - \sum_{l=0}^{r-1} \binom{r}{l} (2^{-q_j-1}M_{j+1})^{t+l}
\end{aligned}
$$

Then,

$$
\begin{aligned}
&\sum_{k=1}^{q_1} a^{k-1}\Bigg(\Big(1+\Big\lfloor \frac{n-1}{2^k}\Big\rfloor\Big)^r \Big\lfloor \frac{n-1}{2^k}+\frac{1}{2}\Big\rfloor^t - \Big\lfloor \frac{n-1}{2^k}+\frac{1}{2}\Big\rfloor^r \Big\lfloor \frac{n-1}{2^k}\Big\rfloor^t\Bigg) \\
&\qquad = \sum_{k=1}^{q_1} a^{k-1} \sum_{l=0}^{r-1} \binom{r}{l} (2^{-k}M_1)^{t+l} = \frac{1}{a}\sum_{l=0}^{r-1} \binom{r}{l} M_1^{t+l} \sum_{k=1}^{q_1} (2^{-(t+l)}a)^k = (*)
\end{aligned}
$$

Now, if $\ell \notin \{0, \dots, r-1\}$, then $2^{-(t+l)}a \neq 1$ for every $l = 0, \dots, r-1$ and hence

$$
(*) = \sum_{l=0}^{r-1} \binom{r}{l} M_1^{t+l} \cdot \frac{1-(2^{-(t+l)}a)^{q_1}}{2^{t+l}-a}
$$

while if $\ell \in \{0, \dots, r-1\}$, then

$$
\begin{aligned}
(*) &= \frac{1}{a}\sum_{\substack{l=0\\ l\neq\ell}}^{r-1} \binom{r}{l} M_1^{t+l} \sum_{k=1}^{q_1} (2^{-(t+l)}a)^k + \frac{1}{a}\binom{r}{\ell} M_1^{t+\ell} \sum_{k=1}^{q_1} 1^k \\
&= \sum_{\substack{l=0\\ l\neq\ell}}^{r-1} \binom{r}{l} M_1^{t+l} \cdot \frac{1-(2^{-(t+l)}a)^{q_1}}{2^{t+l}-a} + \frac{1}{a}\binom{r}{\ell} M_1^{t+\ell} q_1
\end{aligned}
$$

We shall summarize these two cases in a single expression by writing

$$
(*) = \sum_{\substack{l=0\\ l\neq\ell}}^{r-1} \binom{r}{l} M_1^{t+l} \cdot \frac{1-(2^{-(t+l)}a)^{q_1}}{2^{t+l}-a} + \delta_\ell \cdot \frac{1}{a}\binom{r}{\ell} M_1^{t+\ell} q_1. \tag{32}
$$

Notice that if $r = 0$, then $(*) = 0$ and, since in this case $\delta_\ell = 0$, the right-hand side expression of (32) is indeed 0. Without making use of δ_ℓ, (32) would not be true when $r = 0$. In the next sum we shall apply a similar argument to split each sum $\sum_{l=0}^{r-1}$ into a sum $\sum_{\substack{l=0\\ l\neq\ell}}^{r-1}$ and a term that must only be considered when $r > 0$ and $\ell \in \{0, \dots, r-1\}$, and hence it appears multiplied by δ_ℓ.

For every $j = 1, \dots, s-1$:

$$\sum_{k=q_j+1}^{q_{j+1}} a^{k-1}\left(\left(1+\left\lfloor\frac{n-1}{2^k}\right\rfloor\right)^r \left\lfloor\frac{n-1}{2^k}+\frac{1}{2}\right\rfloor^t - \left\lfloor\frac{n-1}{2^k}+\frac{1}{2}\right\rfloor^r \left\lfloor\frac{n-1}{2^k}\right\rfloor^t\right)$$

$$= \sum_{k=q_j+2}^{q_{j+1}} a^{k-1}\sum_{l=0}^{r-1}\binom{r}{l}(2^{-k}M_{j+1})^{t+l} + a^{q_j}\sum_{i=0}^{r+t-1}\binom{r+t}{i}(2^{-q_j-1}M_{j+1})^i$$

$$- a^{q_j}\sum_{l=0}^{r-1}\binom{r}{l}(2^{-q_j-1}M_{j+1})^{t+l}$$

$$= \frac{1}{a}\sum_{l=0}^{r-1}\binom{r}{l}M_{j+1}^{t+l}\sum_{k=q_j+1}^{q_{j+1}}(2^{-(t+l)}a)^k + \frac{1}{a}\sum_{i=0}^{r+t-1}\binom{r+t}{i}M_{j+1}^i(2^{-i}a)^{q_j+1}$$

$$- \frac{2}{a}\sum_{l=0}^{r-1}\binom{r}{l}M_{j+1}^{t+l}(2^{-(t+l)}a)^{q_j+1}$$

$$= \frac{1}{a}\sum_{\substack{l=0\\ l\neq\ell}}^{r-1}\binom{r}{l}M_{j+1}^{t+l}\sum_{k=q_j+1}^{q_{j+1}}(2^{-(t+l)}a)^k + \delta_\ell\frac{1}{a}\binom{r}{\ell}M_{j+1}^{t+\ell}\sum_{k=q_j+1}^{q_{j+1}}1^k$$

$$+ \frac{1}{a}\sum_{i=0}^{r+t-1}\binom{r+t}{i}M_{j+1}^i(2^{-i}a)^{q_j+1} - \frac{2}{a}\sum_{l=0}^{r-1}\binom{r}{l}M_{j+1}^{t+l}(2^{-(t+l)}a)^{q_j+1}$$

$$= \sum_{\substack{l=0\\ l\neq\ell}}^{r-1}\binom{r}{l}M_{j+1}^{t+l}\cdot\frac{(2^{-(t+l)}a)^{q_j}-(2^{-(t+l)}a)^{q_{j+1}}}{2^{t+l}-a} + \delta_\ell\frac{1}{a}\binom{r}{\ell}M_{j+1}^{t+\ell}(q_{j+1}-q_j)$$

$$+ \frac{1}{a}\sum_{i=0}^{r+t-1}\binom{r+t}{i}M_{j+1}^i(2^{-i}a)^{q_j+1} - \frac{2}{a}\sum_{l=0}^{r-1}\binom{r}{l}M_{j+1}^{t+l}(2^{-(t+l)}a)^{q_j+1}$$

Then (and using $q_0 = 0$)

$$\widehat{y}_n = \sum_{k=1}^{q_s} a^{k-1}\left(\left(1+\left\lfloor\frac{n-1}{2^k}\right\rfloor\right)^r \left\lfloor\frac{n-1}{2^k}+\frac{1}{2}\right\rfloor^t - \left\lfloor\frac{n-1}{2^k}+\frac{1}{2}\right\rfloor^r \left\lfloor\frac{n-1}{2^k}\right\rfloor^t\right)$$

$$= \sum_{j=0}^{s-1}\sum_{k=q_j+1}^{q_{j+1}} a^{k-1}\left(\left(1+\left\lfloor\frac{n-1}{2^k}\right\rfloor\right)^r \left\lfloor\frac{n-1}{2^k}+\frac{1}{2}\right\rfloor^t - \left\lfloor\frac{n-1}{2^k}+\frac{1}{2}\right\rfloor^r \left\lfloor\frac{n-1}{2^k}\right\rfloor^t\right)$$

$$= \delta_\ell\cdot\frac{1}{a}\binom{r}{\ell}M_1^{t+\ell}q_1 + \sum_{\substack{l=0\\ l\neq\ell}}^{r-1}\binom{r}{l}M_1^{t+l}\cdot\frac{1-(2^{-(t+l)}a)^{q_1}}{2^{t+l}-a}$$

$$+ \sum_{j=1}^{s-1}\Bigg[\delta_\ell\cdot\frac{1}{a}\binom{r}{\ell}M_{j+1}^{t+\ell}(q_{j+1}-q_j) + \frac{1}{a}\sum_{i=0}^{r+t-1}\binom{r+t}{i}M_{j+1}^i(2^{-i}a)^{q_j+1}$$

$$+ \sum_{\substack{l=0\\ l\neq\ell}}^{r-1}\binom{r}{l}M_{j+1}^{t+l}\cdot\frac{(2^{-(t+l)}a)^{q_j}-(2^{-(t+l)}a)^{q_{j+1}}}{2^{t+l}-a} - \frac{2}{a}\sum_{l=0}^{r-1}\binom{r}{l}M_{j+1}^{t+l}(2^{-(t+l)}a)^{q_j+1}\Bigg]$$

$$= \delta_\ell\cdot\frac{1}{a}\binom{r}{\ell}\sum_{j=0}^{s-1}M_{j+1}^{t+\ell}(q_{j+1}-q_j) + \sum_{\substack{l=0\\ l\neq\ell}}^{r-1}\frac{\binom{r}{l}}{2^{t+l}-a}\sum_{j=0}^{s-1}M_{j+1}^{t+l}\cdot\left((2^{-(t+l)}a)^{q_j}-(2^{-(t+l)}a)^{q_{j+1}}\right)$$

$$+ \sum_{i=0}^{r+t-1}\binom{r+t}{i}\sum_{j=1}^{s-1}2^{-i}M_{j+1}^i(2^{-i}a)^{q_j} - 2\sum_{i=t}^{r+t-1}\binom{r}{i-t}\sum_{j=1}^{s-1}2^{-i}M_{j+1}^i(2^{-i}a)^{q_j} \tag{33}$$

Now,

$$
\begin{aligned}
&\sum_{j=0}^{s-1}((2^{-(t+l)}a)^{q_j} - (2^{-(t+l)}a)^{q_{j+1}})M_{j+1}^{t+l} \\
&\quad = M_1^{t+l} + \sum_{j=1}^{s-1}(2^{-(t+l)}a)^{q_j}(M_{j+1}^{t+l} - M_j^{t+l}) - (2^{-(t+l)}a)^{q_s}M_s^{t+l} \\
&\quad = (n-1)^{t+l} + \sum_{j=1}^{s-1}(2^{-(t+l)}a)^{q_j}(M_{j+1}^{t+l} - (M_{j+1} + 2^{q_j})^{t+l}) - (2^{-(t+l)}a)^{q_s}(2^{q_s})^{t+l} \\
&\quad = (n-1)^{t+l} - a^{q_s} - \sum_{j=1}^{s-1}(2^{-(t+l)}a)^{q_j}\sum_{i=0}^{t+l-1}\binom{t+l}{i}M_{j+1}^{i}2^{(t+l-i)q_j} \\
&\quad = (n-1)^{t+l} - a^{q_s} - \sum_{i=0}^{t+l-1}\binom{t+l}{i}\sum_{j=1}^{s-1}M_{j+1}^{i}(2^{-i}a)^{q_j} \qquad (34)
\end{aligned}
$$

Using this in Eqn. (33), we obtain

$$
\begin{aligned}
\widehat{y}_n &= \delta_\ell \cdot \frac{1}{a}\binom{r}{\ell}\sum_{j=0}^{s-1}M_{j+1}^{t+\ell}(q_{j+1} - q_j) \\
&\quad + \sum_{\substack{l=0\\ l\neq\ell}}^{r-1}\frac{\binom{r}{l}}{2^{t+l}-a}\Big((n-1)^{t+l} - a^{q_s} - \sum_{i=0}^{t+l-1}\binom{t+l}{i}\sum_{j=1}^{s-1}M_{j+1}^{i}(2^{-i}a)^{q_j}\Big) \\
&\quad + \sum_{i=0}^{r+t-1}\binom{r+t}{i}\sum_{j=1}^{s-1}2^{-i}M_{j+1}^{i}(2^{-i}a)^{q_j} - 2\sum_{i=t}^{r+t-1}\binom{r}{i-t}\sum_{j=1}^{s-1}2^{-i}M_{j+1}^{i}(2^{-i}a)^{q_j} \\
&= \delta_\ell \cdot \frac{1}{a}\binom{r}{\ell}\sum_{j=0}^{s-1}M_{j+1}^{t+\ell}(q_{j+1} - q_j) + \sum_{\substack{l=0\\ l\neq\ell}}^{r-1}\frac{\binom{r}{l}}{2^{t+l}-a}\big((n-1)^{t+l} - a^{q_s}\big) \\
&\quad - \sum_{\substack{l=0\\ l\neq\ell}}^{r-1}\sum_{i=0}^{t+l-1}\frac{\binom{r}{l}\binom{t+l}{i}}{2^{t+l}-a}S_{n-1}^{(0,i)} + \sum_{i=0}^{r+t-1}\binom{r+t}{i}2^{-i}S_{n-1}^{(0,i)} - 2\sum_{i=t}^{r+t-1}\binom{r}{i-t}2^{-i}S_{n-1}^{(0,i)} \\
&= \delta_\ell \cdot \frac{1}{a}\binom{r}{\ell}\sum_{j=0}^{s-1}M_{j+1}^{t+\ell}(q_{j+1} - q_j) + \sum_{\substack{l=0\\ l\neq\ell}}^{r-1}\frac{\binom{r}{l}}{2^{t+l}-a}\big((n-1)^{t+l} - a^{q_s}\big) \\
&\quad - \sum_{i=0}^{r+t-1}\sum_{\substack{l=i-t+1\\ l\neq\ell}}^{r-1}\frac{\binom{r}{l}\binom{t+l}{i}}{2^{t+l}-a}S_{n-1}^{(0,i)} + \sum_{i=0}^{r+t-1}\binom{r+t}{i}2^{-i}S_{n-1}^{(0,i)} - 2\sum_{i=0}^{r+t-1}\binom{r}{i-t}2^{-i}S_{n-1}^{(0,i)} \\
&= \delta_\ell \cdot \frac{1}{a}\binom{r}{\ell}\sum_{j=0}^{s-1}M_{j+1}^{t+\ell}(q_{j+1} - q_j) + \sum_{\substack{l=0\\ l\neq\ell}}^{r-1}\frac{\binom{r}{l}}{2^{t+l}-a}\big((n-1)^{t+l} - a^{q_s}\big) \\
&\quad + \sum_{i=0}^{r+t-1}\Big(2^{-i}\binom{r+t}{i} - 2^{-i+1}\binom{r}{i-t} - \sum_{\substack{l=i-t+1\\ l\neq\ell}}^{r-1}\frac{\binom{r}{l}\binom{t+l}{i}}{2^{t+l}-a}\Big)S_{n-1}^{(0,i)}
\end{aligned}
$$

as we claimed. □

Proposition 10. *For every $n \geqslant 1$:*

$$\alpha_n^{(d,m)} = \frac{1}{2(m+1)} \sum_{i=1}^{s} \sum_{j=0}^{m} \binom{m+1}{j} B_j 2^j M_i^{m+1-j} \big(T(d, q_i, a2^{j-m}) - T(d, q_{i-1}, a2^{j-m})\big)$$
$$+ \sum_{i=1}^{s-1} q_i^d (a2^{-m})^{q_i} (n - M_i) M_{i+1}^m - T(d, q_{s_n}(n), 2a) \cdot \delta_{m=0}$$

with $\delta_{m=0} = 1$ if $m = 0$ and $\delta_{m=0} = 0$ if $m > 0$.

Proof. When $m = 0$, by Lemma 3 we have that

$$\begin{aligned}
\alpha_n^{(d,0)} &= \sum_{k=1}^{n-1} \sum_{j=1}^{s_k - 1} q_j(k)^d a^{q_j(k)} = \sum_{k=1}^{n-1} \sum_{j=1}^{s_k} q_j(k)^d a^{q_j(k)} - \sum_{k=1}^{n-1} q_{s_k}(k)^d a^{q_{s_k}(k)} \\
&= \sum_{i=1}^{s_n} 2^{q_i(n)-1} T(d, q_i(n), a) + \sum_{i=1}^{s_n} q_i(n)^d a^{q_i(n)} (n - M_i(n)) \\
&\qquad - T(d, q_{s_n}(n), 2a) - (n - 2^{q_{s_n}(n)}) q_{s_n}(n)^d a^{q_{s_n}(n)} \\
&= \sum_{i=1}^{s_n} 2^{q_i(n)-1} T(d, q_i(n), a) + \sum_{i=1}^{s_n - 1} q_i(n)^d a^{q_i(n)} (n - M_i(n)) - T(d, q_{s_n}(n), 2a)
\end{aligned} \tag{35}$$

This agrees with the expression for $\alpha_n^{(d,m)}$ given in the statement when $m = 0$, because

$$\begin{aligned}
&\frac{1}{2} \sum_{i=1}^{s_n} M_i(n) \big(T(d, q_i(n), a) - T(d, q_{i-1}(n), a)\big) + \sum_{i=1}^{s_n - 1} q_i(n)^d a^{q_i(n)} (n - M_i(n)) \\
&\quad = \frac{1}{2} \sum_{i=1}^{s_n} T(d, q_i(n), a)(M_i(n) - M_{i+1}(n))\big) + \sum_{i=1}^{s_n - 1} q_i(n)^d a^{q_i(n)} (n - M_i(n)) \\
&\quad = \sum_{i=1}^{s_n} T(d, q_i(n), a) 2^{q_i(n)-1} + \sum_{i=1}^{s_n - 1} q_i(n)^d a^{q_i(n)} (n - M_i(n)) \\
&\quad = \alpha_n^{(d,0)} + T(d, q_{s_n}(n), 2a)
\end{aligned}$$

Let us consider now the case $m > 0$. We first prove that, for every $m \in \mathbb{N}_{\geqslant 1}$, $d \in \mathbb{N}$, $M \in \mathbb{N}_{\geqslant 1}$, and $0 \leqslant l \leqslant q_1(M)$,

$$\alpha_{M+2^l}^{(d,m)} = \alpha_M^{(d,m)} + \alpha_{2^l}^{(d,m)} + \sum_{p=0}^{m-1} \binom{m}{p} M^{m-p} \gamma_l^{(d,p,m)} + 2^l S_M^{(d,m)} \tag{36}$$

Indeed, let $M = \sum_{i=1}^{s} 2^{q_i}$, with $s \geqslant 1$ and $q_s > \cdots > q_1 > 0$, and let $0 \leqslant l \leqslant q_1$, so that $s_M = s$ and $q_i(M) = q_i$ for each $i = 1, \ldots, s$. Then,

$$\begin{aligned}
\alpha_{M+2^l}^{(d,m)} &= \sum_{k=1}^{M-1} \sum_{i=1}^{s_k - 1} q_i(k)^d (a2^{-m})^{q_i(k)} M_{i+1}(k)^m + \sum_{k=M}^{M+2^l-1} \sum_{i=1}^{s_k - 1} q_i(k)^d (a2^{-m})^{q_i(k)} M_{i+1}(k)^m \\
&= \alpha_M^{(d,m)} + \sum_{k=M}^{M+2^l-1} \sum_{i=1}^{s_k - 1} q_i(k)^d (a2^{-m})^{q_i(k)} M_{i+1}(k)^m
\end{aligned}$$

where

$$\begin{aligned}
&\sum_{k=M}^{M+2^l-1}\sum_{i=1}^{s_k-1} q_i(k)^d (a2^{-m})^{q_i(k)} M_{i+1}(k)^m \\
&\quad = \sum_{k=M}^{M+2^l-1}\sum_{i=1}^{s_k-s} q_i(k)^d (a2^{-m})^{q_i(k)} M_{i+1}(k)^m + \sum_{k=M}^{M+2^l-1}\sum_{i=s_k-s+1}^{s_k-1} q_i(k)^d (a2^{-m})^{q_i(k)} M_{i+1}(k)^m \\
&\quad = \sum_{k=M}^{M+2^l-1}\sum_{i=1}^{s_k-s} q_i(k)^d (a2^{-m})^{q_i(k)} (M_{i+1}(k-M)+M)^m + \sum_{k=M}^{M+2^l-1}\sum_{i=1}^{s-1} q_i^d (a2^{-m})^{q_i} M_{i+1}(M)^m \\
&\quad = \sum_{k=0}^{2^l-1}\sum_{i=1}^{s_k} q_i(k)^d (a2^{-m})^{q_i(k)} (M_{i+1}(k)+M)^m + 2^l \sum_{i=1}^{s-1} q_i^d (a2^{-m})^{q_i} M_{i+1}(M)^m \\
&\quad = \sum_{k=1}^{2^l-1}\sum_{i=1}^{s_k} q_i(k)^d (a2^{-m})^{q_i(k)} \sum_{p=0}^{m}\binom{m}{p} M_{i+1}(k)^p M^{m-p} + 2^l S_M^{(d,m)} \\
&\quad = \sum_{p=0}^{m-1}\binom{m}{p} M^{m-p} \sum_{k=1}^{2^l-1}\sum_{i=1}^{s_k} q_i(k)^d (a2^{-m})^{q_i(k)} M_{i+1}(k)^p + \sum_{k=1}^{2^l-1}\sum_{i=1}^{s_k} q_i(k)^d (a2^{-m})^{q_i(k)} M_{i+1}(k)^m \\
&\qquad + 2^l S_M^{(d,m)} \\
&\quad = \sum_{p=0}^{m-1}\binom{m}{p} M^{m-p}\gamma_l^{(d,p,m)} + \alpha_{2^l}^{(d,m)} + 2^l S_M^{(d,m)}
\end{aligned}$$

yielding Eqn. (36).

From this Eqn. (36) it is easy to deduce that, for every $m \in \mathbb{N}_{\geqslant 1}$ and for every $l \geqslant 0$,

$$\alpha_{2^l}^{(d,m)} = \frac{2^{ml+l-1}}{m+1}\sum_{p=0}^{m}\binom{m+1}{p} 2^{-p(l-1)} B_p \cdot T(d, l-1, 2^{p-m}a) \tag{37}$$

Indeed, taking $M = 2^l$ in Eqn. (36) and recalling that $S_{2^l}^{(d,m)} = 0$ because $s_{2^l} = 1$), we obtain

$$\alpha_{2^{l+1}}^{(d,m)} = 2\alpha_{2^l}^{(d,m)} + \sum_{p=0}^{m-1}\binom{m}{p} 2^{l(m-p)}\gamma_l^{(d,p,m)}$$

The solution of this recurrence with $\alpha_{2^0}^{(d,m)} = \alpha_1^{(d,m)} = 0$ is

$$\alpha_{2^l}^{(d,m)} = \sum_{k=1}^{l-1} 2^{l-k-1}\sum_{p=0}^{m-1}\binom{m}{p} 2^{k(m-p)}\gamma_k^{(d,p,m)}$$

Then, using Lemma 5,

$$
\begin{aligned}
&\alpha_{2^l}^{(d,m)} \\
&= \sum_{k=1}^{l-1} 2^{l-k-1} \sum_{p=0}^{m-1} \binom{m}{p} 2^{k(m-p)} \frac{a^{k-1} 2^{-(m-1)(k-1)+pk}}{p+1} \sum_{t=1}^{k-1} (k-t-1)^d (a^{-1} 2^{m-p-1})^t (B_{p+1}(2^t) - B_{p+1}) \\
&\quad + \sum_{k=1}^{l-1} 2^{l-k-1} 2^{km} (a 2^{-(m-1)})^{k-1} (k-1)^d \\
&= \frac{2^{m+l-2}}{m+1} \sum_{t=1}^{l-2} \left[\Big(\sum_{p=0}^{m-1} \binom{m+1}{p+1} 2^{(m-p-1)t} (B_{p+1}(2^t) - B_{p+1}) \Big) \Big(\sum_{k=t+1}^{l-1} (k-t-1)^d a^{k-t-1} \Big) \right] \\
&\quad + 2^{m+l-2} \sum_{k=1}^{l-1} (k-1)^d a^{k-1} \\
&= \frac{2^{m+l-2}}{m+1} \sum_{t=1}^{l-2} \left[\Big(\sum_{p=1}^{m} \binom{m+1}{p} 2^{(m-p)t} (B_p(2^t) - B_p) \Big) \Big(\sum_{k=0}^{l-t-2} k^d a^k \Big) \right] + 2^{m+l-2} \sum_{k=0}^{l-2} k^d a^k \\
&= \frac{2^{m+l-2}}{m+1} \sum_{t=0}^{l-2} \left[\Big(\sum_{p=0}^{m} \binom{m+1}{p} 2^{(m-p)t} (B_p(2^t) - B_p) \Big) T(d, l-t-1, a) \right]
\end{aligned}
$$

by Eqn. (9). Now,

$$
\begin{aligned}
&\sum_{p=0}^{m} \binom{m+1}{p} 2^{(m-p)t} (B_p(2^t) - B_p) \\
&\qquad = 2^{-t} \sum_{p=0}^{m} \binom{m+1}{p} 2^{t(m+1-p)} B_p(2^t) - \sum_{p=0}^{m} \binom{m+1}{p} 2^{(m-p)t} B_p \\
&\qquad = 2^{-t} (B_{m+1}(2^{t+1}) - B_{m+1}(2^t)) - \sum_{p=0}^{m} \binom{m+1}{p} 2^{(m-p)t} B_p \\
&\qquad = 2^{-t} \sum_{p=0}^{m+1} \binom{m+1}{p} 2^{(m+1-p)(t+1)} B_p - 2^{-t} \sum_{p=0}^{m+1} \binom{m+1}{p} 2^{(m+1-p)t} B_p - \sum_{p=0}^{m} \binom{m+1}{p} 2^{(m-p)t} B_p \\
&\qquad = 2 \sum_{p=0}^{m} \binom{m+1}{p} (2^{(m-p)(t+1)} - 2^{(m-p)t}) B_p
\end{aligned}
$$

and therefore

$$
\begin{aligned}
\alpha_{2^l}^{(d,m)} &= \frac{2^{m+l-2}}{m+1} \sum_{t=0}^{l-2} \left[\Big(2 \sum_{p=0}^{m} \binom{m+1}{p} (2^{(m-p)(t+1)} - 2^{(m-p)t}) B_p \Big) T(d, l-t-1, a) \right] \\
&= \frac{2^{m+l-1}}{m+1} \sum_{p=0}^{m} \binom{m+1}{p} B_p \cdot \left[\sum_{t=0}^{l-2} 2^{(m-p)(t+1)} T(d, l-t-1, a) - \sum_{t=0}^{l-2} 2^{(m-p)t} T(d, l-t-1, a) \right] \\
&= \frac{2^{m+l-1}}{m+1} \sum_{p=0}^{m} \binom{m+1}{p} B_p \cdot \left[\sum_{t=1}^{l-1} 2^{(m-p)t} T(d, l-t, a) - \sum_{t=0}^{l-2} 2^{(m-p)t} T(d, l-t-1, a) \right] \\
&= \frac{2^{m+l-1}}{m+1} \sum_{p=0}^{m} \binom{m+1}{p} B_p \cdot \Bigg[\sum_{t=1}^{l-1} 2^{(m-p)t} (l-t-1)^d a^{l-t-1} + \sum_{t=1}^{l-2} 2^{(m-p)t} T(d, l-t-1, a) \\
&\qquad\qquad - \sum_{t=0}^{l-2} 2^{(m-p)t} T(d, l-t-1, a) \Bigg] \\
&= \frac{2^{m+l-1}}{m+1} \sum_{p=0}^{m} \binom{m+1}{p} B_p \cdot \left[\sum_{t=1}^{l-1} 2^{(m-p)t} (l-t-1)^d a^{l-t-1} - T(d, l-1, a) \right] \\
&= \frac{2^{m+l-1}}{m+1} \sum_{p=0}^{m} \binom{m+1}{p} B_p \cdot \Big(\sum_{t=1}^{l-1} 2^{(m-p)t} (l-t-1)^d a^{l-t-1} \Big) \qquad \text{(by Eqn. (7))} \\
&= \frac{2^{ml+l-1}}{m+1} \sum_{p=0}^{m} \binom{m+1}{p} 2^{-p(l-1)} B_p \cdot \Big(\sum_{t=1}^{l-1} (l-t-1)^d (2^{p-m} a)^{l-t-1} \Big) \\
&= \frac{2^{ml+l-1}}{m+1} \sum_{p=0}^{m} \binom{m+1}{p} 2^{-p(l-1)} B_p \cdot \Big(\sum_{k=0}^{l-2} k^d (2^{p-m} a)^{k} \Big) \\
&= \frac{2^{ml+l-1}}{m+1} \sum_{p=0}^{m} \binom{m+1}{p} 2^{-p(l-1)} B_p \cdot T(d, l-1, 2^{p-m} a)
\end{aligned}
$$

as we claimed in Eqn. (37).

The time has come to prove the stated general expression for $\alpha_n^{(d,m)}$. To simplify the notations, let $s = s_n$ and $q_i = q_i(n)$ and $M_i = M_i(n) = \sum_{h=i}^{s} 2^{q_h}$, for every $i = 1, \ldots, s$. Then, Eqn. (36) and a simple argument by induction implies that

$$
\begin{aligned}
\alpha_n^{(d,m)} &= \sum_{k=1}^{s} \alpha_{2^{q_k}}^{(d,m)} + \sum_{p=0}^{m-1} \binom{m}{p} \sum_{i=1}^{s-1} M_{i+1}^{m-p} \gamma_{q_i}^{(d,p,m)} + \sum_{k=1}^{s-1} 2^{q_k} S_{M_{k+1}}^{(d,m)} \\
&= \sum_{k=1}^{s} \alpha_{2^{q_k}}^{(d,m)} + \sum_{p=0}^{m-1} \binom{m}{p} \sum_{i=1}^{s-1} M_{i+1}^{m-p} \gamma_{q_i}^{(d,p,m)} + \sum_{k=1}^{s-1} 2^{q_k} \sum_{i=k+1}^{s-1} q_i^d (a 2^{-m})^{q_i} M_{i+1}^m \\
&= \sum_{k=1}^{s} \alpha_{2^{q_k}}^{(d,m)} + \sum_{p=0}^{m-1} \binom{m}{p} \sum_{i=1}^{s-1} M_{i+1}^{m-p} \gamma_{q_i}^{(d,p,m)} + \sum_{i=2}^{s-1} q_i^d (a 2^{-m})^{q_i} M_{i+1}^m \sum_{k=1}^{i-1} 2^{q_k} \\
&= \sum_{k=1}^{s} \alpha_{2^{q_k}}^{(d,m)} + \sum_{p=0}^{m-1} \binom{m}{p} \sum_{i=1}^{s-1} M_{i+1}^{m-p} \gamma_{q_i}^{(d,p,m)} + \sum_{i=1}^{s-1} q_i^d (a 2^{-m})^{q_i} (n - M_i) M_{i+1}^m \qquad (38)
\end{aligned}
$$

where, with the convention that $M^0_{s+1}=1$,

$$\sum_{k=1}^{s}\alpha_{2^{q_k}}^{(d,m)}=\sum_{k=1}^{s}\frac{2^{mq_k+q_k-1}}{m+1}\sum_{p=0}^{m}\binom{m+1}{p}2^{-p(q_k-1)}B_p\cdot T(d,q_k-1,2^{p-m}a)$$
$$=\sum_{k=1}^{s}\frac{2^{mq_k+q_k-1}}{m+1}\sum_{p=0}^{m}\binom{m+1}{p}2^{-p(q_k-1)}B_p\cdot\Big(\sum_{t=0}^{q_k-2}t^d(2^{p-m}a)^t\Big)$$
$$=\frac{2^m}{m+1}\sum_{p=0}^{m}\binom{m+1}{p}B_p\cdot\Big(\sum_{k=1}^{s}2^{(m-p+1)(q_k-1)}\sum_{t=0}^{q_k-2}t^d(2^{p-m}a)^t\Big)$$
$$=\frac{2^m}{m+1}\sum_{p=0}^{m-1}\binom{m+1}{m+1}\binom{m+1}{p}B_p\cdot\Big(\sum_{k=1}^{s}M_{k+1}^{m-m}2^{(m-p+1)(q_k-1)}\sum_{t=0}^{q_k-2}t^d(2^{p-m}a)^t\Big)$$
$$+2^{m-1}B_m\cdot\Big(\sum_{k=1}^{s}2^{q_k}\sum_{t=0}^{q_k-2}t^da^t\Big)$$

and, by Lemma 5, setting $\delta_{q_1\geqslant 1}=\min\{q_1,1\}$

$$\sum_{p=0}^{m-1}\binom{m}{p}\sum_{i=1}^{s-1}M_{i+1}^{m-p}\gamma_{q_i}^{(d,p,m)}=\sum_{p=0}^{m-1}\binom{m}{p}\sum_{i=1}^{s}M_{i+1}^{m-p}\gamma_{q_i}^{(d,p,m)}$$
$$=\sum_{p=0}^{m-1}\binom{m}{p}\sum_{i=1}^{s}M_{i+1}^{m-p}\frac{a^{q_i-1}2^{-(m-1)(q_i-1)+pq_i}}{p+1}\sum_{t=1}^{q_i-1}(q_i-t-1)^d(a^{-1}2^{m-p-1})^t(B_{p+1}(2^t)-B_{p+1})$$
$$+M_2^m(a2^{-(m-1)})^{q_1-1}(q_1-1)^d\delta_{q_1\geqslant 1}+\sum_{i=2}^{s}M_{i+1}^m(a2^{-(m-1)})^{q_i-1}(q_i-1)^d$$
$$=\frac{1}{m+1}\sum_{p=0}^{m-1}\binom{m+1}{p+1}2^p\sum_{i=1}^{s}M_{i+1}^{m-p}\sum_{t=1}^{q_i-1}(q_i-t-1)^d(2^{p-m+1}a)^{q_i-t-1}(B_{p+1}(2^t)-B_{p+1})$$
$$+M_2^m(a2^{-(m-1)})^{q_1-1}(q_1-1)^d\delta_{q_1\geqslant 1}+\sum_{i=2}^{s}M_{i+1}^m(a2^{-(m-1)})^{q_i-1}(q_i-1)^d$$
$$=\frac{1}{m+1}\sum_{p=0}^{m-1}\binom{m+1}{p+1}2^p\sum_{i=1}^{s}M_{i+1}^{m-p}\sum_{t=0}^{q_i-2}t^d(2^{p-m+1}a)^t(B_{p+1}(2^{q_i-t-1})-B_{p+1})$$
$$+M_2^m(a2^{-(m-1)})^{q_1-1}(q_1-1)^d\delta_{q_1\geqslant 1}+\sum_{i=2}^{s}M_{i+1}^m(a2^{-(m-1)})^{q_i-1}(q_i-1)^d$$
$$=\frac{1}{m+1}\sum_{p=0}^{m-1}\binom{m+1}{p+1}2^p\sum_{i=1}^{s}M_{i+1}^{m-p}\sum_{t=0}^{q_i-2}t^d2^{(p-m+1)t}a^t\sum_{j=0}^{p}\binom{p+1}{j}2^{(p+1-j)(q_i-t-1)}B_j$$
$$+M_2^m(a2^{-(m-1)})^{q_1-1}(q_1-1)^d\delta_{q_1\geqslant 1}+\sum_{i=2}^{s}M_{i+1}^m(a2^{-(m-1)})^{q_i-1}(q_i-1)^d$$
$$=\frac{1}{m+1}\sum_{j=0}^{m-1}\sum_{p=j}^{m-1}\binom{m+1}{p+1}\binom{p+1}{j}2^p\sum_{i=1}^{s}M_{i+1}^{m-p}2^{(p+1-j)(q_i-1)}\sum_{t=0}^{q_i-2}t^d(a2^{-(m-j)})^tB_j$$
$$+M_2^m(a2^{-(m-1)})^{q_1-1}(q_1-1)^d\delta_{q_1\geqslant 1}+\sum_{i=2}^{s}M_{i+1}^m(a2^{-(m-1)})^{q_i-1}(q_i-1)^d$$

Thus, returning back to (38) (and still with the convention that $M_{s+1}^0 = 1$), we have

$$\begin{aligned}
\alpha_n^{(d,m)} &= \frac{2^m}{m+1}\sum_{j=0}^{m-1}\binom{m+1}{m+1}\binom{m+1}{j}\Big(\sum_{i=1}^{s} M_{i+1}^{m-m}2^{(m-j+1)(q_i-1)}\sum_{t=0}^{q_i-2} t^d(2^{j-m}a)^t\Big)B_j \\
&\quad + 2^{m-1}B_m\cdot\Big(\sum_{i=1}^{s}2^{q_i}\sum_{t=0}^{q_i-2}t^d a^t\Big) \\
&\quad + \frac{1}{m+1}\sum_{j=0}^{m-1}\sum_{p=j}^{m-1}\binom{m+1}{p+1}\binom{p+1}{j}2^p\sum_{i=1}^{s}M_{i+1}^{m-p}2^{(p+1-j)(q_i-1)}\sum_{t=0}^{q_i-2}t^d(a2^{-(m-j)})^tB_j \\
&\quad + M_2^m(a2^{-(m-1)})^{q_1-1}(q_1-1)^d\delta_{q_1\geqslant 1} + \sum_{i=2}^{s}M_{i+1}^m(a2^{-(m-1)})^{q_i-1}(q_i-1)^d \\
&\quad + \sum_{i=1}^{s-1}q_i^d(a2^{-m})^{q_i}(n-M_i)M_{i+1}^m \\
&= \frac{1}{m+1}\sum_{j=0}^{m-1}\sum_{p=j}^{m}\binom{m+1}{p+1}\binom{p+1}{j}2^p\sum_{i=1}^{s}M_{i+1}^{m-p}2^{(p+1-j)(q_i-1)}\sum_{t=0}^{q_i-2}t^d(a2^{-(m-j)})^tB_j \\
&\quad + 2^{m-1}B_m\cdot\Big(\sum_{i=1}^{s}2^{q_i}\sum_{t=0}^{q_i-2}t^d a^t\Big) \\
&\quad + M_2^m(a2^{-(m-1)})^{q_1-1}(q_1-1)^d\delta_{q_1\geqslant 1} + \sum_{i=2}^{s}M_{i+1}^m(a2^{-(m-1)})^{q_i-1}(q_i-1)^d \\
&\quad + \sum_{i=1}^{s-1}q_i^d(a2^{-m})^{q_i}(n-M_i)M_{i+1}^m \\
&= \frac{1}{m+1}\sum_{j=0}^{m}\sum_{p=j}^{m}\binom{m+1}{p+1}\binom{p+1}{j}2^p\sum_{i=1}^{s}M_{i+1}^{m-p}2^{(p+1-j)(q_i-1)}\sum_{t=0}^{q_i-2}t^d(a2^{-(m-j)})^tB_j \\
&\quad + M_2^m(a2^{-(m-1)})^{q_1-1}(q_1-1)^d\delta_{q_1\geqslant 1} + \sum_{i=2}^{s}M_{i+1}^m(a2^{-(m-1)})^{q_i-1}(q_i-1)^d \\
&\quad + \sum_{i=1}^{s-1}q_i^d(a2^{-m})^{q_i}(n-M_i)M_{i+1}^m
\end{aligned} \tag{39}$$

Now notice that, for every $i = 1, \dots, s$

$$\begin{aligned}
&\frac{1}{m+1}\sum_{j=0}^{m}\sum_{p=j}^{m}\binom{m+1}{p+1}\binom{p+1}{j}2^pM_{i+1}^{m-p}2^{(p+1-j)(q_i-1)}(q_i-1)^d(a2^{-(m-j)})^{q_i-1}B_j \\
&\quad = \frac{1}{m+1}(q_i-1)^d a^{q_i-1}\sum_{p=0}^{m}\binom{m+1}{p+1}M_{i+1}^{m-p}2^{(p+1-m)(q_i-1)+p}\sum_{j=0}^{p}\binom{p+1}{j}B_j \\
&\quad = \frac{1}{m+1}(q_i-1)^d a^{q_i-1}(m+1)M_{i+1}^m 2^{-(m-1)(q_i-1)} \qquad \text{(by Eqn. (7))} \\
&\quad = (q_i-1)^d M_{i+1}^m(a2^{-(m-1)})^{q_i-1}
\end{aligned}$$

Therefore, if $q_1 \geqslant 1$, (39) becomes

$$\begin{aligned}
\alpha_n^{(d,m)} &= \frac{1}{m+1}\sum_{j=0}^{m}\sum_{p=j}^{m}\binom{m+1}{p+1}\binom{p+1}{j}2^p\sum_{i=1}^{s}M_{i+1}^{m-p}2^{(p+1-j)(q_i-1)}\sum_{t=0}^{q_i-2}t^d(a2^{-(m-j)})^tB_j \\
&\quad+\sum_{i=1}^{s}M_{i+1}^{m}(a2^{-(m-1)})^{q_i-1}(q_i-1)^d+\sum_{i=1}^{s-1}q_i^d(a2^{-m})^{q_i}(n-M_i)M_{i+1}^m \\
&= \frac{1}{m+1}\sum_{j=0}^{m}\sum_{p=j}^{m}\binom{m+1}{p+1}\binom{p+1}{j}2^p\sum_{i=1}^{s}M_{i+1}^{m-p}2^{(p+1-j)(q_i-1)}\sum_{t=0}^{q_i-1}t^d(a2^{-(m-j)})^tB_j \\
&\quad+\sum_{i=1}^{s-1}q_i^d(a2^{-m})^{q_i}(n-M_i)M_{i+1}^m
\end{aligned}$$

while, if $q_1 = 0$,

$$\begin{aligned}
\alpha_n^{(d,m)} &= \frac{1}{m+1}\sum_{j=0}^{m}\sum_{p=j}^{m}\binom{m+1}{p+1}\binom{p+1}{j}2^p\sum_{i=1}^{s}M_{i+1}^{m-p}2^{(p+1-j)(q_i-1)}\sum_{t=0}^{q_i-2}t^d(a2^{-(m-j)})^tB_j \\
&\quad+\sum_{i=2}^{s}M_{i+1}^{m}(a2^{-(m-1)})^{q_i-1}(q_i-1)^d+\sum_{i=1}^{s-1}q_i^d(a2^{-m})^{q_i}(n-M_i)M_{i+1}^m \\
&= \frac{1}{m+1}\sum_{j=0}^{m}\sum_{p=j}^{m}\binom{m+1}{p+1}\binom{p+1}{j}2^p\sum_{i=2}^{s}M_{i+1}^{m-p}2^{(p+1-j)(q_i-1)}\sum_{t=0}^{q_i-2}t^d(a2^{-(m-j)})^tB_j \\
&\quad+\sum_{i=2}^{s}M_{i+1}^{m}(a2^{-(m-1)})^{q_i-1}(q_i-1)^d+\sum_{i=1}^{s-1}q_i^d(a2^{-m})^{q_i}(n-M_i)M_{i+1}^m \\
&= \frac{1}{m+1}\sum_{j=0}^{m}\sum_{p=j}^{m}\binom{m+1}{p+1}\binom{p+1}{j}2^p\sum_{i=2}^{s}M_{i+1}^{m-p}2^{(p+1-j)(q_i-1)}\sum_{t=0}^{q_i-1}t^d(a2^{-(m-j)})^tB_j \\
&\quad+\sum_{i=1}^{s-1}q_i^d(a2^{-m})^{q_i}(n-M_i)M_{i+1}^m \\
&= \frac{1}{m+1}\sum_{j=0}^{m}\sum_{p=j}^{m}\binom{m+1}{p+1}\binom{p+1}{j}2^p\sum_{i=1}^{s}M_{i+1}^{m-p}2^{(p+1-j)(q_i-1)}\sum_{t=0}^{q_i-1}t^d(a2^{-(m-j)})^tB_j \\
&\quad+\sum_{i=1}^{s-1}q_i^d(a2^{-m})^{q_i}(n-M_i)M_{i+1}^m
\end{aligned}$$

So, in summary, for every $n \geqslant 1$,

$$\begin{aligned}
\alpha_n^{(d,m)} &= \frac{1}{m+1}\sum_{j=0}^{m}\sum_{p=j}^{m}\binom{m+1}{p+1}\binom{p+1}{j}2^p\sum_{i=1}^{s}M_{i+1}^{m-p}2^{(p+1-j)(q_i-1)}\sum_{t=0}^{q_i-1}t^d(a2^{-(m-j)})^tB_j \\
&\quad+\sum_{i=1}^{s-1}q_i^d(a2^{-m})^{q_i}(n-M_i)M_{i+1}^m \qquad (40)
\end{aligned}$$

It remains to simplify the first term in the right-hand side expression:

$$\begin{aligned}
&\sum_{j=0}^{m}\sum_{p=j}^{m}\binom{m+1}{p+1}\binom{p+1}{j}2^p\sum_{i=1}^{s}M_{i+1}^{m-p}2^{(p+1-j)(q_i-1)}\sum_{t=0}^{q_i-1}t^d(a2^{-(m-j)})^tB_j\\
&\quad=\sum_{i=1}^{s}\sum_{t=0}^{q_i-1}t^da^t\sum_{j=0}^{m}\sum_{p=j}^{m}\binom{m+1}{j}\binom{m+1-j}{m-p}M_{i+1}^{m-p}2^{(p+1-j)q_i+j-1-(m-j)t}B_j\\
&\quad=\sum_{i=1}^{s}\sum_{t=0}^{q_i-1}t^da^t\sum_{j=0}^{m}\sum_{p=0}^{m-j}\binom{m+1}{j}\binom{m+1-j}{p}M_{i+1}^{p}2^{(m-p+1-j)q_i+j-1-(m-j)t}B_j\\
&\quad=\sum_{i=1}^{s}\sum_{t=0}^{q_i-1}t^da^t\sum_{j=0}^{m}\Big(\sum_{p=0}^{m-j}\binom{m+1-j}{p}M_{i+1}^{p}2^{(m+1-j-p)q_i}\Big)\binom{m+1}{j}2^{j-1-(m-j)t}B_j\\
&\quad=\sum_{i=1}^{s}\sum_{t=0}^{q_i-1}t^da^t\sum_{j=0}^{m}(M_i^{m+1-j}-M_{i+1}^{m+1-j})\binom{m+1}{j}2^{j-1-(m-j)t}B_j\\
&\quad=\frac{1}{2}\sum_{j=0}^{m}\binom{m+1}{j}B_j2^j\sum_{i=1}^{s}(M_i^{m+1-j}-M_{i+1}^{m+1-j})T(d,q_i,a2^{j-m})\\
&\quad=\frac{1}{2}\sum_{j=0}^{m}\binom{m+1}{j}B_j2^j\sum_{i=1}^{s}M_i^{m+1-j}\big(T(d,q_i,a2^{j-m})-T(d,q_{i-1},a2^{j-m})\big)
\end{aligned}$$

So, returning back to Eqn. (40), we finally obtain

$$\begin{aligned}
\alpha_n^{(d,m)}=&\frac{1}{2(m+1)}\sum_{i=1}^{s}\sum_{j=0}^{m}\binom{m+1}{j}B_j2^jM_i^{m+1-j}\big(T(d,q_i,a2^{j-m})-T(d,q_{i-1},a2^{j-m})\big)\\
&+\sum_{i=1}^{s-1}q_i^d(a2^{-m})^{q_i}(n-M_i)M_{i+1}^m
\end{aligned}$$

as we claimed. ∎

Remark 11. When $n=2^l$, the formula in Proposition 10 says

$$\begin{aligned}
\alpha_{2^l}^{(d,m)}&=\frac{1}{2(m+1)}\sum_{j=0}^{m}\binom{m+1}{j}B_j2^{j+(m+1-j)l}T(d,l,a2^{j-m})\\
&=\frac{2^{(m+1)l-1}}{m+1}\sum_{j=0}^{m}\binom{m+1}{j}B_j2^{-j(l-1)}T(d,l,a2^{j-m})
\end{aligned}$$

and this expression is equivalent to Eqn. (37),

$$\alpha_{2^l}^{(d,m)}=\frac{2^{(m+1)l-1}}{m+1}\sum_{j=0}^{m}\binom{m+1}{j}B_j2^{-j(l-1)}T(d,l-1,a2^{j-m}),$$

because

$$\sum_{j=0}^{m}\binom{m+1}{j}B_j2^{-j(l-1)}(a2^{j-m})^{l-1}=2^{-m(l-1)}\sum_{j=0}^{m}\binom{m+1}{j}B_j=0$$
