# Supplementary material for: Explicit solution of divide-and-conquer dividing by a half recurrences with polynomial independent term
Source: PLoS One. 2022 Nov 17;17(11):e0274448. doi: 10.1371/journal.pone.0274448 (PMC9671444; doi:10.1371/journal.pone.0274448)
Supplement: Attachment — Submitted filename: responsetoreviewers.pdf [file pone.0274448.s002.pdf]

Department of Mathematics
and Computer Science
University of the Balearic Islands
E-07122 Palma de Mallorca
Spain

August 1, 2022

Staff Editors
PLOS ONE

Dear Dr Ashwani Kumar,

Thank you very much for the review on the paper PONE-D-22-15094 "Explicit solution of divide-and-conquer dividing by a half recurrences with polynomial independent term". We have revised our paper to take into account your comments and those of the reviewers. Below you will find the detailed replies to these comments. We have also taken the opportunity to fix some typos and grammatical errors. Finally, let me apologize for the delay in sending the new version of the paper, I was in charge of it and I had an unusually very busy month of July.

Yours sincerely, Francesc Rosselló

Journal Requirements

1) Please ensure that your manuscript meets PLOS ONE's style requirements [...]
   Done
2) Concerning the funding information [...].
   We have removed from the Acknowledgements Section the funding information. The correct Funding Statement is given in the cover letter.
3) Please review your reference list to ensure that it is complete and correct.
   Done. We have removed an "orphan" reference, and we have added the new references [2], [4], [12], [25], [32], [38], [40], [44] that are cited in the tex added in this new version.

Reviewer 1

1) The abstract should contain answers to the following questions: What problem was studied and why is it important? Add a few applications of the current elaborated problem, and avoid citation in the abstract. Don't used equation in the abstract, remove it if feasible.
   We have slightly rewritten the Abstract following this suggestion. We believe the displayed equation must remain, to explain exactly what kind of equations we solve.
2) The motivation and gap of study is not really highlighted in the last paragraph of the introduction section. The paragraph should elaborate more on the importance, and address/highlight the research contributions.
   See the next item
3) The originality of the paper needs to be stated clearly. It is of important to have sufficient results to justify the novelty of a high-quality journal paper. The Introduction should make a compelling case for why the study is useful along with a clear statement of its novelty or originality by providing relevant information and providing answers to basic questions such as: What is already known in the open literature? What is missing (i.e., research gaps)? What needs to be done, why and how? Clear statements of the novelty of the work should also appear briefly in the Abstract

and Conclusions sections.
We have expanded the information on these topics in the introduction.

4) Check all the equations for possible typo mistakes.
We have checked carefully the equations both in the main text and the Supplementary Material file and still found some typos. We have highlighted the corrections in red.

5) Must write real application of elaborated problem.
We have included explanations of the interest of the examples included in the Results section. We have replaced former example 7, which did not add anything new to former example 6, by the new example 4, related to the Takagi curve and yielding a new result.

6) Write future direction of the current study.
We have expanded the information on it in the Conclusions section

Reviewer 2

1) What is new in the model equations and why these are considered?
Divide-and-conquer dividing by a half with polynomial independent term are not new at all. The novelty is that we know how to solve them.

2) Is it necessary to restrict yourselves to the case when the toll function is a polynomial?
In this paper yes, but as we mention (and now emphasize) in the Conclusions, we believe that the same technique could be used to obtain explicit solutions of other types of equations.

3) When will explicit solution zero?
We have answered this question in Remark 2

4) Correlate your work with the earlier ones and provide distinction of your work.
Done in paragraphs added in the Introduction
